# Assessment of stability of a Himalayan road cut slope with varying degrees of weathering: A finite-element-model-based approach

**DOI:** 10.1016/j.heliyon.2020.e05297

**Published:** 2020-11-16

**Authors:** Gbétoglo Charles Komadja, Sarada Prasad Pradhan, Amulya Ratna Roul, Babatunde Adebayo, Jean Baptiste Habinshuti, Luc Adissin Glodji, Azikiwe Peter Onwualu

**Affiliations:** a Department of Materials Science and Engineering, African University of Science and Technology, Abuja, Nigeria; b Department of Earth Sciences, Indian Institute of Technology Roorkee, Roorkee, India; c Department of Mining Engineering, Federal University of Technology Akure, Akure, Nigeria; d Department of Earth Sciences, University of Abomey-Calavi, Abomey-Calavi, Benin

**Keywords:** Geology, Earth sciences, Natural hazards, Landslides, Slope stability, Finite element method, Strength reduction factor, Slope geometry, Weathering

## Abstract

Slope stability assessment is essential for safe and sustainable development widely applied in mining, civil, and environmental engineering projects around the world. This study aimed to conduct a stability analysis of a selected Himalayan road cut slope from two different sections, named sections (A) and (B). The strength reduction factor (SRF) based on the finite element method was used to simulate the slope sections using Phase2 software. A mesh pattern of six node triangle elements was used during the numerical simulation. The Mohr-Coulomb parameters and other inputs used in the numerical modelling of the investigated slope were estimated by different geotechnical tests, namely, the direct shear test, density analysis test, rock hardness test, and Brazilian test. The results indicated that the critical SRF of the completely weathered slope profile section (A), with a relatively low overall angle, was found to be 1.25, which is approximately 50% lower than the value obtained in the moderately to highly weathered profile section (B), equal to 2.53. These results are in agreement with other published studies, which revealed that the geometry of a slope influences the weathering grade, which in turn destabilizes the slope. The results of this study will help in engineering slope design considering the influence of weathering.

## 1 Introduction

Landslides are a natural phenomenon that involves the displacement (falling, toppling, sliding, and flowing) of materials that form a slope. This is mainly caused by the influence of gravity in response to natural and anthropogenic activities and causes damage to the surface morphology and surrounding structure, as well as fatalities in the living community exposed along the slope. Fatalities and other disastrous effects induced by landslides have been reported in the literature (Froude and Petley, 2018; Nirupama, 2015; Pradhan et al., 2019; Skilodimou et al., 2018; Vishal et al., 2017). Landslides depend on several factors, such as geology, geomorphology, climate conditions, anthropogenic activities, and earthquakes (Dai et al., 2001; Ermias et al., 2017; Raghuvanshi, 2019; Skilodimou et al., 2018; Summa et al., 2015; Tang et al., 2017; Thiery et al., 2019). The stability of a slope is evaluated by comparing the shear strength (cohesion and friction angle) defined as the ratio of resisting forces (working load) to driving forces (collapse load). The slope is considered stable if the resisting force is greater than the driving force. The ratio of resisting forces to driving forces is known as the factor of safety (FoS) which characterises the stability of the slope (Bishop, 1955; Bushira et al., 2018; Pradhan et al., 2014; Raghuvanshi, 2019; Renani and Martin, 2020). Theoretically, a slope with FoS less than one is unstable and vulnerable to failure, whereas a slope with FoS greater than one resists failure. A reduction in the shear strength augments the instability of a slope. Kaczmarek and Popielski (2019) studied the impact of a number of variables on the slope equilibrium and found that the angle of internal friction is one of the most sensitive parameters for the slope stability. There have been several attempts at understanding the factors affecting the shear strength parameters of a rock/soil material. Xiao-Song et al. (2018) characterised the shear strength parameters of a rock mass/soil and indicated that a greater quality rock mass provides higher shear strength parameters. The influence of the alteration of a rock mass on rock engineering parameters has been reported by Everall and Sanislav (2018). Similarly, Jesus et al. (2018) reported that the strength of a rock formation decreases as the degree of weathering and thickness of the residual-formed soil increases. Arvanitidis et al. (2019) investigated the relationship between the peak friction angle of the soil and the grain size distribution and observed that the effective peak friction angle increases as the coarse-to-fines ratio increases. Thongkhao et al. (2015) highlighted the influence of the slope gradient and weathering grade on landslide occurrence in northern Thailand. The results of their study show that the weathering grade impacts the engineering properties of the residual soil from the parent rock and significantly destabilises the equilibrium state of the slope. Many other studies have highlighted the reduction in the inherent shear strength of material constituting a slope (Azañón et al., 2010; Hajdarwish et al., 2013; Kumar et al., 2017; Li, 2018; Pradhan and Siddique, 2020; Siddique and Pradhan, 2018; Yalcin, 2011; Yates et al., 2018).

The Himalayan mountain chain is one of the most unique attractive geological features on the earth. Its rough topography is the product of a collision between the Indian plate and Eurasian plate. Ongoing tectonic activities make this area more interesting for geological studies. Landslide events are one of the most common phenomena in this area, as in any mountainous area (Pradhan et al., 2018; Qing-zhao et al., 2019). In each monsoon, as well as during many seismic events, landslides are recorded along roads, hilly villages, and towns. Along National Highway 7 (NH-7), a number of landslides have been recorded in the recent past and are most often correlated with the disintegration of the rock mass and unplanned excavation of the road cut slopes.

The portion from Shivpuri to Devprayag is one of the most vulnerable areas for landslides. Siddique and Pradhan (2018) studied the stability analysis of road cut debris slopes along the former National Highway 58 (NH-58), currently renamed NH-7. They compared the stability of a number of slopes located in different sites with different geological features, using two different modelling techniques, namely, the limit equilibrium (LE) method and the strength reduction factor (SRF) method based on the finite element method (FEM). The findings of their research were in line with other research studies (Digvijay et al., 2017; Griffiths and Lane, 1999), revealing that FEM–SRF is capable of simulating a slope more accurately and automatically provides the FoS for the critical slip surface without any assumptions. In the SRF method, the shear strength parameters (cohesion and friction angle) of the materials forming the slope are progressively reduced until the instability state of the slope is reached and the SRF is calculated, which is equivalent to the FoS (Bushira et al., 2018; Siddique and Pradhan, 2018; Tsige et al., 2020; Yang et al., 2010; Zienkiewicz et al., 1977). In this work, a road cut slope near Shivpuri is demarcated, and FEM–SRF is used to evaluate the stability of the slope from different sections. Although the equilibrium condition of the road cut debris slopes is studied by Siddique and Pradhan (2018), the effect of the geometry and geological features of the slopes are combined, and the specific effect of these parameters cannot be easily distinguishable. The aim of this study is to evaluate the effective influence of the slope geometry and weathering conditions of the individual specific rock cut slope at different sections, rather than adopting indirect correlations between slopes in different locations and with different geological properties.

## 2 Study area

**Figure 1:**
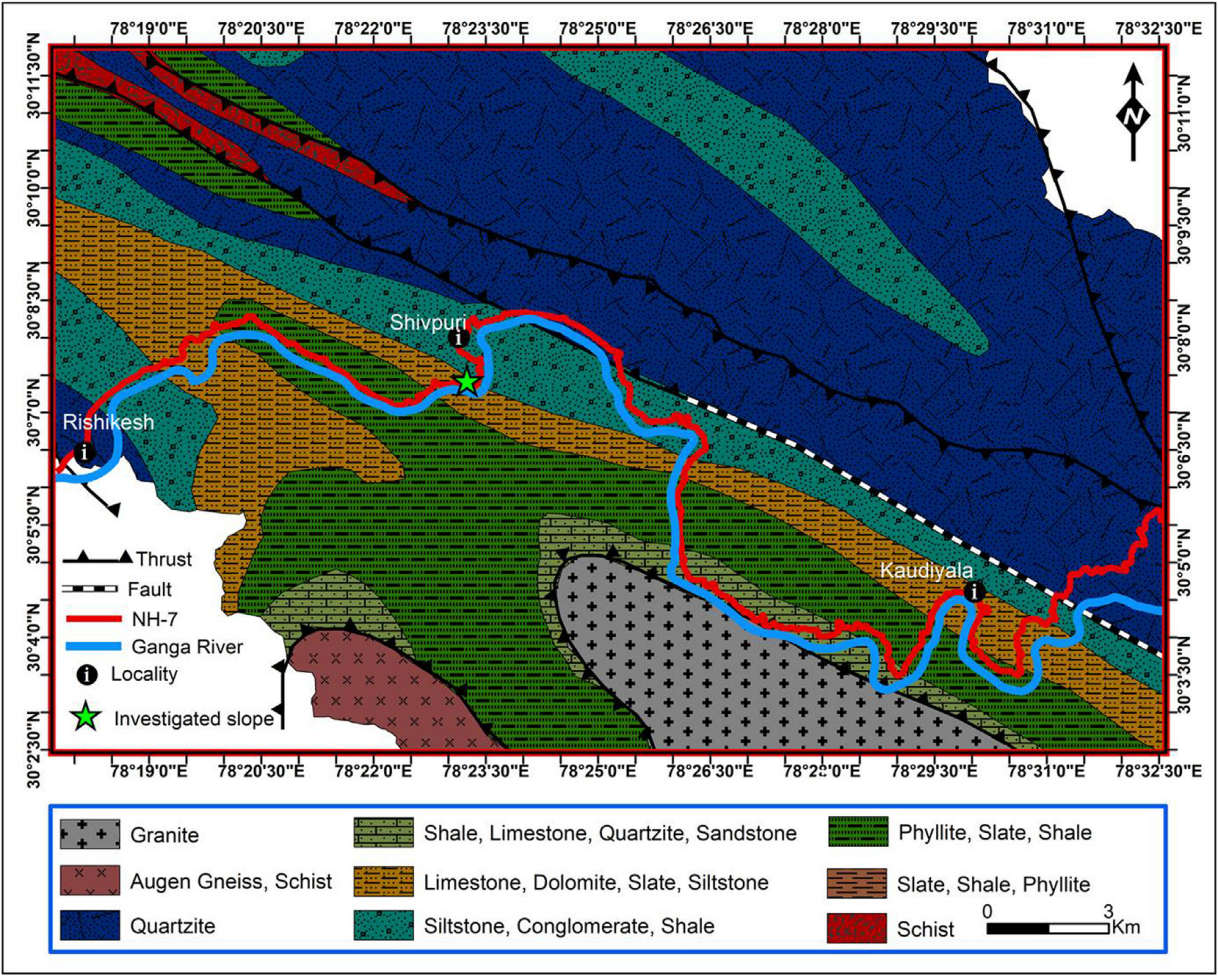
Location of the investigated slope on the geological map of the study area (Valdiya, 1980; modified by Siddique and Pradhan, 2018).

The study area is located in the Lesser Himalayan region, along NH-7, formerly named NH-58. The road is quasi-parallel to the Ganga River in the Garhwal syncline of the outer Lesser Himalayan region (Figure 1). The Lesser Himalayan region is sealed by the main boundary thrust in the south and the main central thrust in the north (Valdiya, 1980, 1983) (Figure 1). Metasedimentary formations are encountered in the Lesser Himalayan (Le Fort, 1975; Pradhan and Siddique, 2020; Valdiya, 1980, 1983). Outcrops of carbonaceous rock bearing limestone, with defined levels of sandstone, quartzite, shale, and phyllitic formations have been reported and identified during the field investigation. These rocks, moderately to completely weathered, lead to the formation of the debris overlying slopes. The thickness of this debris above the bedrock ranges from 1 to 10 m.

## 3 Methodology

### 3.1 Field visit and slope section identification

A detailed field investigation was carried out to measure the geometry of the slope (height and length) and the orientations of structural features (dip and dip direction). A brief description of the lithology and geological features of the study area has been provided. The weathering grade of the slope was assessed based on the visual description, rock discolouration processes, hand scraping on rock masses and hammer, sound of the rock under the impact of a geological hammer, and Schmidt hammer tests (Biondino et al., 2020; Ietto et al., 2018; Jun-Yao et al., 2020; Moses et al., 2014). The determination of the degree of weathering was based on the main weathering classification system, which classifies and characterises the weathering horizon into six classes from fresh/unweathered bedrock (grade I) to residual and colluvial soil (grade VI), as described in Table 1. Two different slope profile sections (A) and (B) of the same slope have been investigated following the bypass of the road (Figure 6). A significant variation in the weathering grade on the slope faces was identified during field investigation. The slope profile Section (A) was covered by a cap of debris, indicating the prominence of weathering at this portion, as compared with section (B). Two representative debris samples were collected on the basis of a visual description of the material overlying the slope at section (A) and loose material from the toe region of the slope, labelled L1S1 and L1S2, respectively.

**Table 1:** Weathering grades of rock and rock masses (Biondino et al., 2020; Hearn, 2011; Mohamad et al., 2008).

Description	Grade	Rock material				Rock mass			
		Colour	Texture	Slaking		Structure		Iron- rich layer	Strength (Schmidt hammer)
				In water	By hand	Condition	Changes		
Sound rock (SR)	I	No changes	Unchanged	Remains as mass	Edges unbroken	100% intact	No changes	None	Exceed 25
Slightly weathered rock (SW)	II						Discolouration along discontinuity		
					Edges can be broken			May exist	
Moderately weathered rock (MW)	III	Slightly discoloured					Iron-rich filling discontinuity		Less than 25
Highly weathered rock (HW)	IV	Completely discoloured		Becomes flakes or small pieces	Becomes flakes or small pieces	>50–75% remains			
Completely weathered rock (CW)	V	Completely changed	Half remains unchanged	Disintegrated	Disintegrated	<25% remains	Completely changed	Normally exist	None
Residual and colluvial soil (S)	VI	Completely changed	Destroyed			100% destroyed		None	

### 3.2 Determination of geotechnical parameters

**Figure 2:**
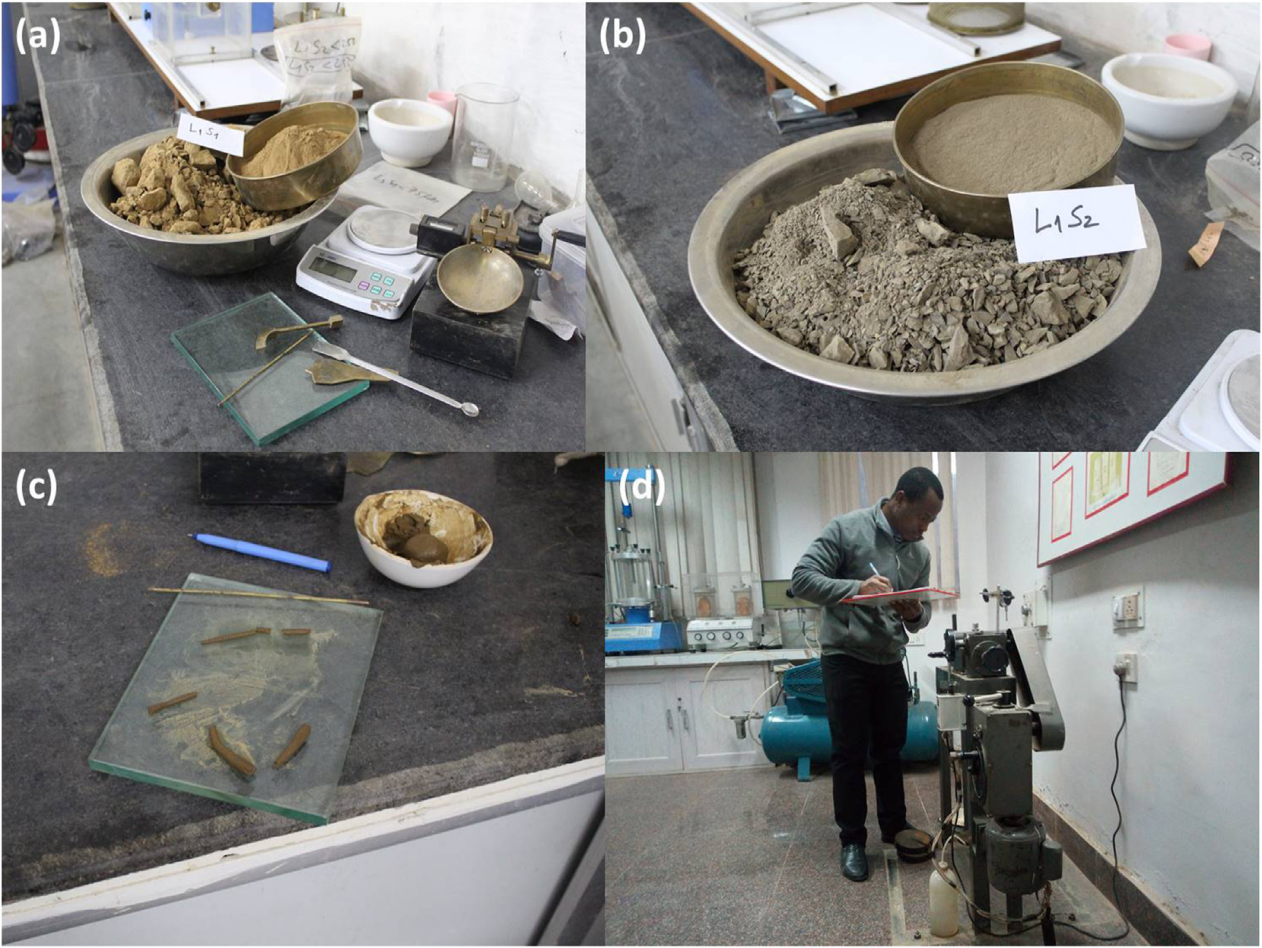
Debris geotechnical properties; (a) debris L1S1 with Atterberg limits test apparatus, (b) debris L1S2, (c) Atterberg limits testing, (d) direct shear strength testing.

Important geotechnical properties of the representative samples were assessed by laboratory experiments in the Department of Earth Sciences, and Civil Engineering at the Indian Institute of Technology Roorkee (IIT Roorkee) as well as in the Geotechnical Engineering laboratory at CSIR-CBRI, Roorkee. Laboratory characterisation included sieve analysis (grain size distribution) of the debris samples following the relevant standard (ASTM D421−85, 2007). The soil fraction passing through sieve number #40 (0.425 mm) was used to evaluate Atterberg limits (liquid limit, plastic limit) and the resultant plasticity index (Figure 2a, b, and c). The density analysis test was performed to estimate the unit weight of the rock mass and debris materials (ASTM D1556/D1556M, 2011), and the Young's modulus of the rock mass was evaluated based on the Schmidt hammer hardness test, following the suggested method of Aydin (2015). The Schmidt hardness test is an ideal, non-destructive, and in-situ technique, which is often employed in many rock mechanics and rock engineering practices. It is the most widely used method to determine several properties, such as the modulus of elasticity (*E*) of the geological materials. In the examination, 20 readings of (L-type) Schmidt hammer rebound were randomly recorded, and the average value of 42.5 was obtained and used to determine the Young's modulus (Table 3) based on the empirical relationship developed by Yagiz (2009) on similar lithology with a very good coefficient of determination (R = 0.92) (Eq. 1)

(Eq. 1) *E* (GPa) = 0.0987 *Hr*^(1.5545)^

where *E* is the Young modulus and *Hr* is the rebound hardness value.

Brazilian test method was used to determine the indirect tensile strength (*Ts*) of the rockmass (Eq. 2) as per the guideline suggested by (ASTM D2936, 2008; ISRM, 1978)

(Eq. 2) $$T_{S}\;\left(MPa\right)=\frac{2P}{\pi Dt}$$

where, *P* represents the load at failure, *D* is the diameter of the test specimen (mm), and *t* is the thickness of the test specimen measured at the center (mm).

The shear strength parameters of the rockmass and the Poisson ratio (ν) of the slope materials were obtained from relevant literature on Himalayan rock formation (Pradhan and Siddique, 2020; Siddique and Pradhan, 2018) assuring the φ–ν inequality (Eq. 3) highlighted by Zheng et al. (2005) for any geological formation in which the shear strength is expressed in terms of cohesion (C) and angle of internal friction (φ) (Mohr–Coulomb's failure criterion). The φ–ν inequality prevents the underestimation of the result and FoS during computation (Zheng et al., 2005)

(Eq. 3) sinφ≥1−2ν

where φ is friction angle and ν represents the Poisson ratio.

**Figure 3:**
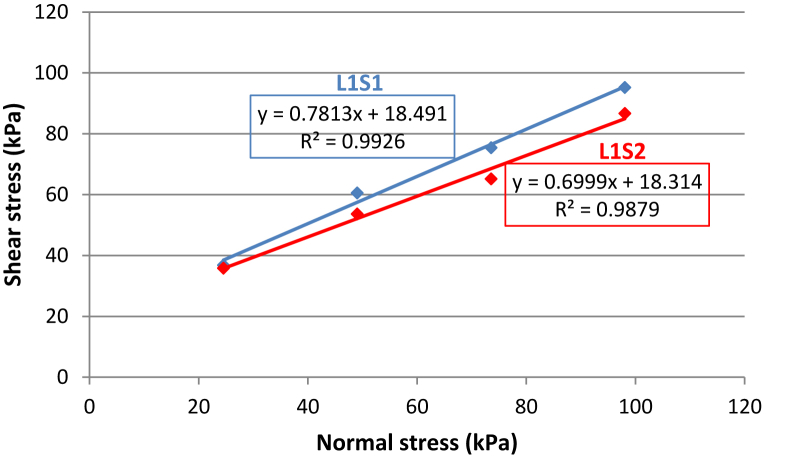
Determination of the shear strength parameters of the debris using the results of direct shear tests.

In this study, the slope material is assumed to act as a Coulomb material, and the shear strength parameters, namely, cohesion (C) and angle of internal friction(φ), were determined from undrained direct shear testing on the specimens (Figure 2 a, b and d) following ASTM standard (ASTM D6528, 2017). The test was conducted under four different normal loads of 0.25 kg/cm^2^, 0.5 kg/cm^2^, 0.75 kg/cm^2^, and 1 kg/cm^2^ and the applied normal stresses ranged from 24.51 kN/m^2^, to 98.06 kPa with a shearing rate of 0.01. The dial gauge reading and shear displacement were recorded for each normal load applied. Then, the shear stress at failure was determined and plotted against the normal stress for each test (Figure 3). Using the generated graph, the cohesion (C) and the angle of internal friction (φ) of the debris was calculated based on the Coulomb failure criterion (Eq. 4)

(Eq. 4) $${\text{τ}}_{\text{f}}=\text{c}+{\text{σ}}_{\text{f}}\;\tan\;\varphi$$

where $\tau_{f}$is the ultimate shear stress at failure; cis the apparent cohesion of the soil sample; $\sigma_{f}$is the normal stress at failure plane; and φ is the angle of internal friction.

### 3.3 Slope stability analysis by finite element modelling

A finite-element-based numerical simulator in the Phase2 software program was used to generate the critical shear SRF, which is equivalent to the FoS. A mesh pattern of six node triangle elements was used during numerical simulation to determine the maximum shear strength at each node.

## 4 Results and discussion

**Figure 4:**
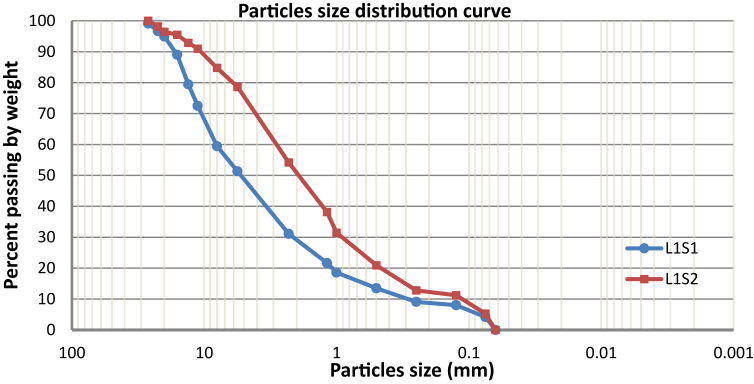
Particle-size distribution curve of the debris materials.

The result of the sieve analysis is presented in Figure 4. The gradation parameters of the soil, namely, the uniformity coefficient (Cu) and the coefficient of curvature (Cc), are tabulated and reported in Table 2. For both samples, the fraction of fines is less than 5%, the uniformity coefficient (Cu) > 6, and the coefficient of curvature lies between 1 < (Cc) < 3, classifying both debris samples as SW, i.e., well-graded sands, gravelly sands, with little or no fines (Selig and Howard, 1984).

**Table 2:** Unified soil classification system (USCS) of the samples and their plasticity index.

Samples	Gradation parameters			Atterberg limits		
	Cu	Cc	D50	Liquid limit	Plastic limit	Plasticity index
L1S1	26.92	1.92	6.39	25.1%	21.49%	3.61
L1S2	10.70	1.24	3.51	23.60%	21.79%	1.81

Cu, uniformity coefficient; Cc, coefficient of curvature; D50, mean particle size.

**Figure 5:**
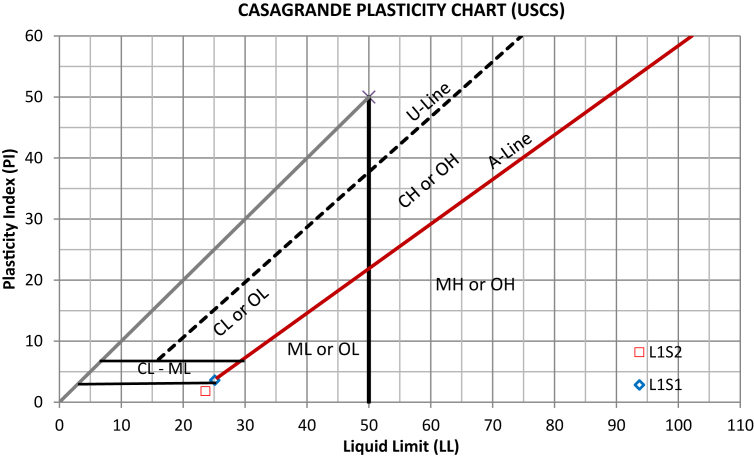
Plot of the debris samples in the Casagrande plasticity chart.

The liquid limit (LL) and plasticity index (PI) of the samples were found to be 25.10% and 3.61% for sample (L1S1) and 23.60% and 1.81% for sample (L1S2), respectively; from the Casagrande plasticity chart presented in Figure 5, both debris are graded silt of low plasticity (ML). As can be seen in Table 2, the gradation parameters of the residual debris soil L1S1 overlying the slope surface at section (A) are slightly greater than the loose, relatively fine material from the surface of the rock mass sampled at the lower portion of the slope. This is in agreement with the result reported by Getahun et al. (2019), indicating that soil with smaller grain size provides the lowest grading coefficient and friction angle. Table 3 presents the parameters used to perform the stability analysis of the slope from both sides (sections A and B. The direct shear test showed a higher friction angle for both samples (38°–35°) with relatively similar cohesion). As shown in Tables 2 and 3, a linear relationship between the soil gradation parameters and strength parameters (friction angle) can be highlighted. The debris L1S1, with a relatively higher plasticity index and soil gradation parameters, exhibited a greater friction angle than debris L1S2 at the surface of the bedrock. Aligned with the result of the study conducted by Igwe et al. (2007), the friction angle of the soil increases with an increase in its physical properties, e.g., the uniformity coefficient (Cu), mean particle size (D50), and unit weight.

**Table 3:** Input parameters used for modelling.

	Cohesion (MPa)	Friction angle (degree)	Unit weight (MN/m^3^)	Tensile strength (MPa)	Young modulus (GPa)	Poisson ratio
**Debris soil parameters**						
L1S1	0.01849	38	0.0203	0	20	0.3∗
L1S2	0.01831	35	0.0171	0	20	0.3∗
**Rock mass parameters**						
Limestone	0.178	32.52	0.0255	3.22	33.547	0.385∗

∗ Value from (Siddique & Pradhan, 2018).

**Figure 6:**
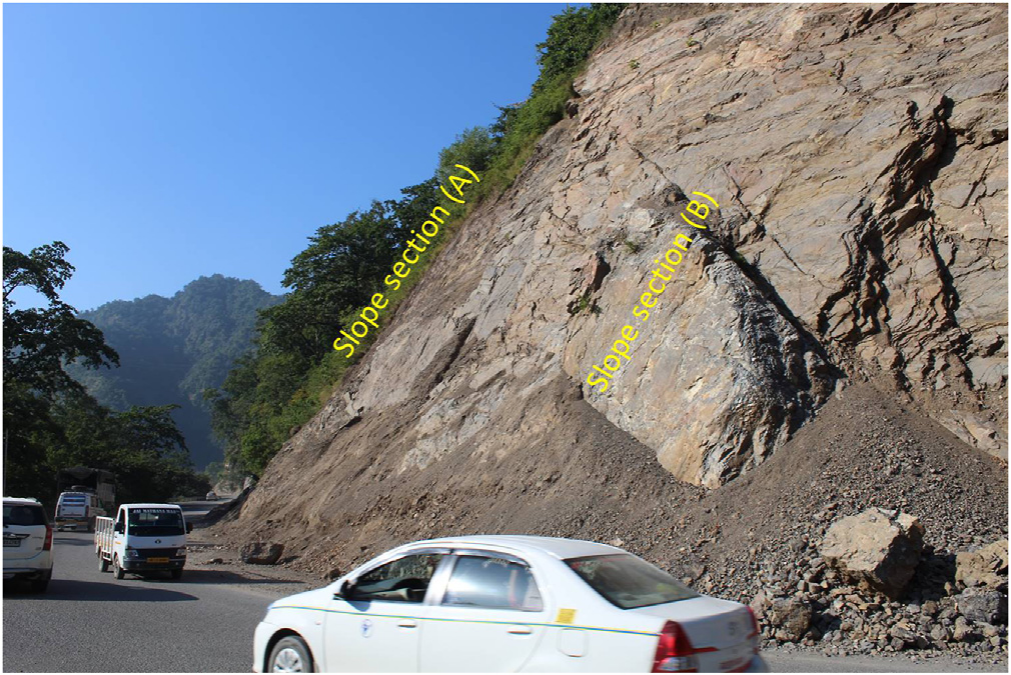
General overview of the investigated slope.

**Figure 7:**
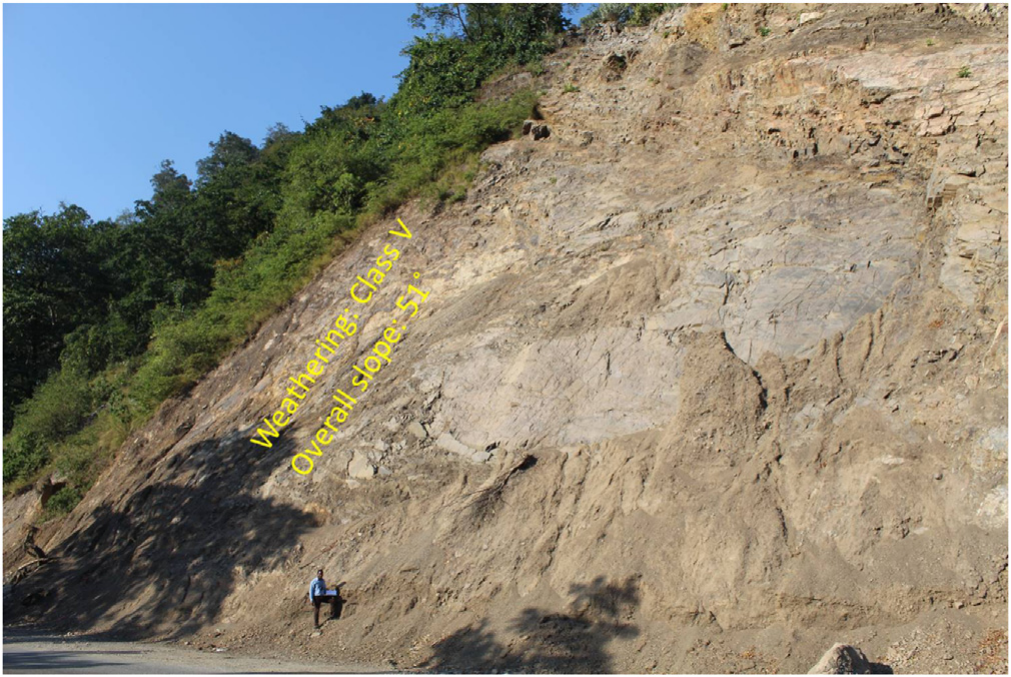
Slope profile section (A) showing the overall slope angle and the weathering grade.

**Figure 8:**
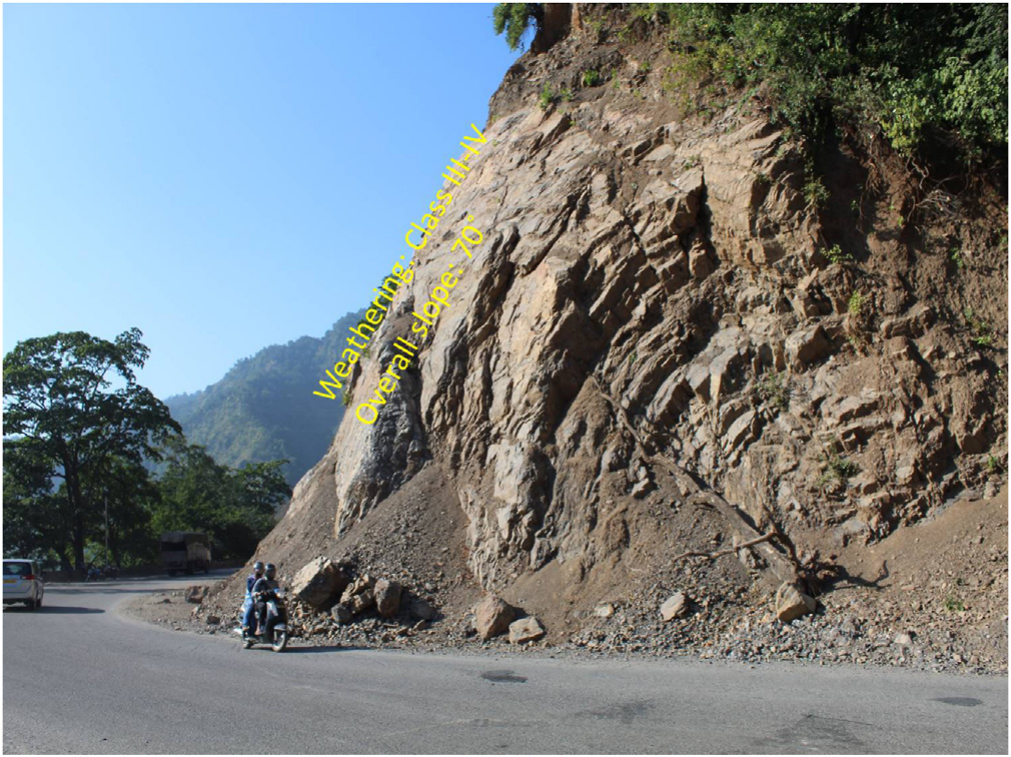
Slope profile section (B) showing the overall slope angle and the weathering grade.

**Figure 9:**
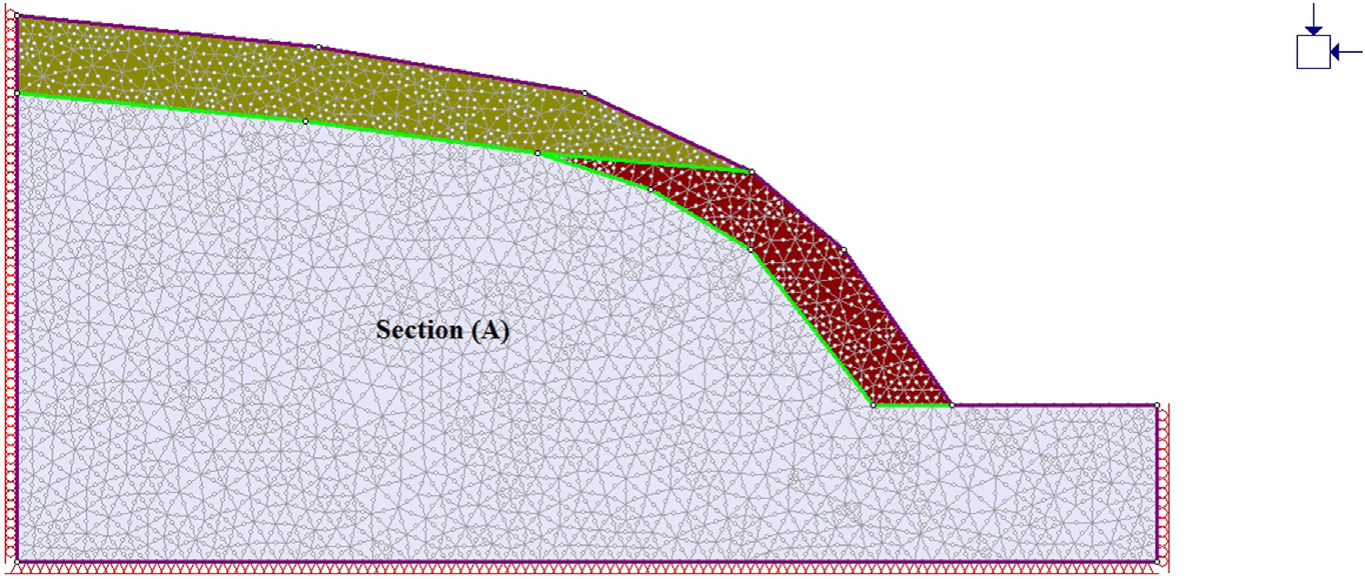
Geometry of the simulated slope along with the debris at section A.

**Figure 10:**
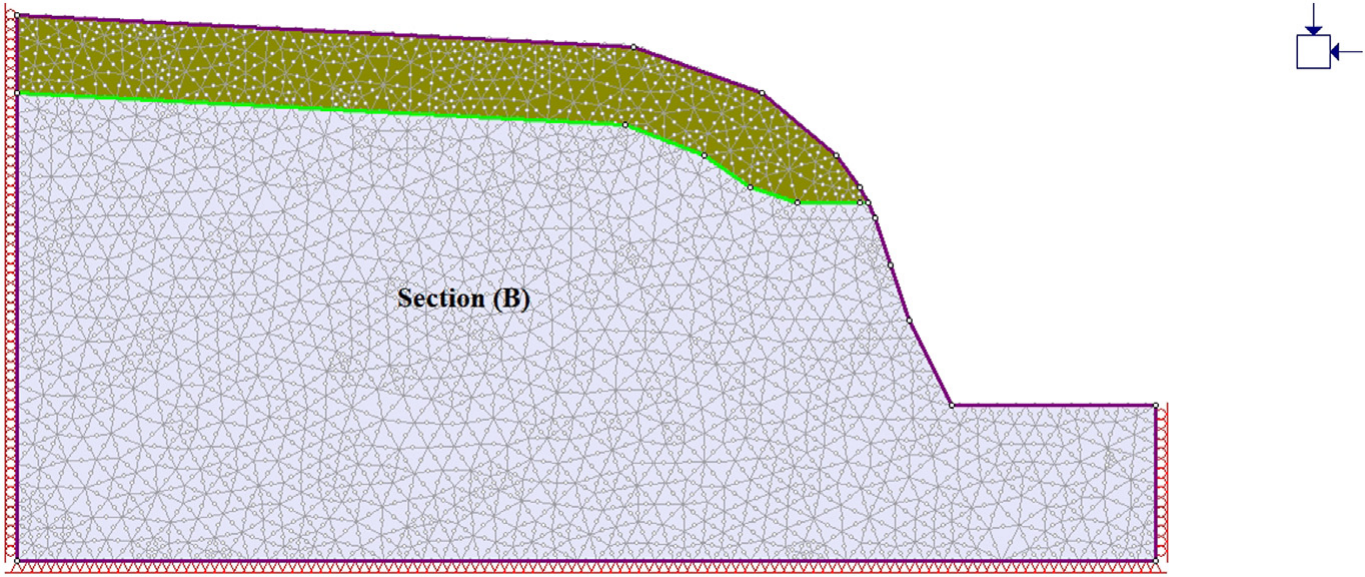
Geometry of the simulated slope along with the debris at section B.

**Figure 11:**
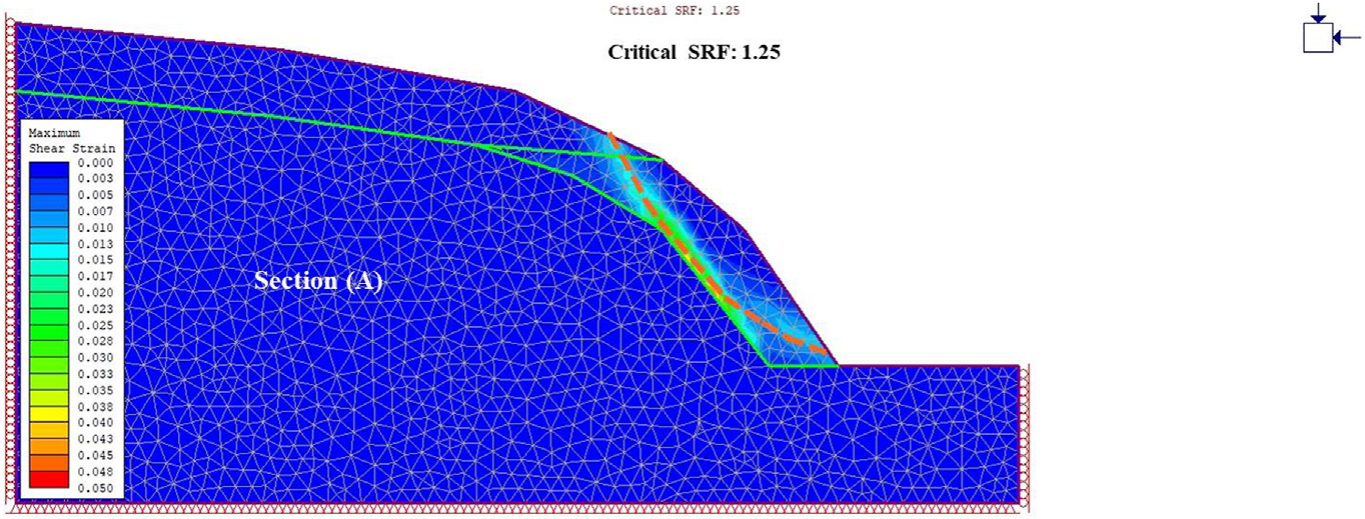
Shear strain dispersion showing a nearly circular failure pattern at section A.

**Figure 12:**
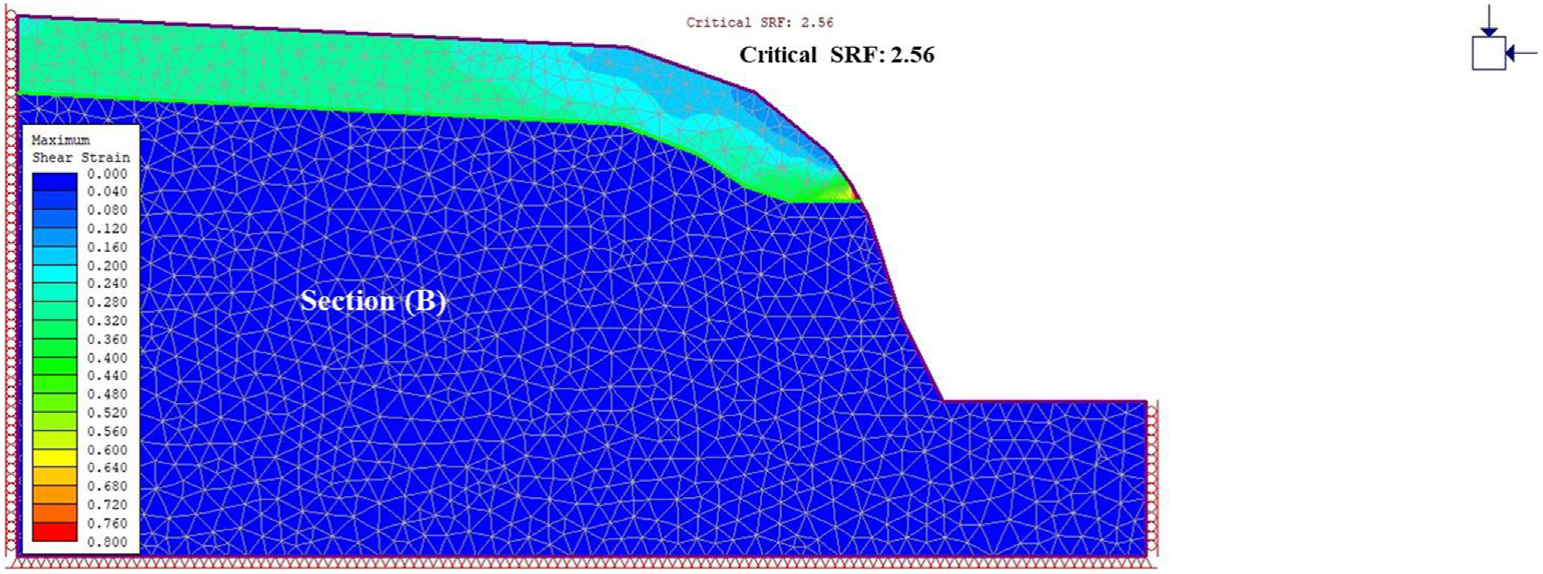
Shear strain dispersion at section B.

**Figure 13:**
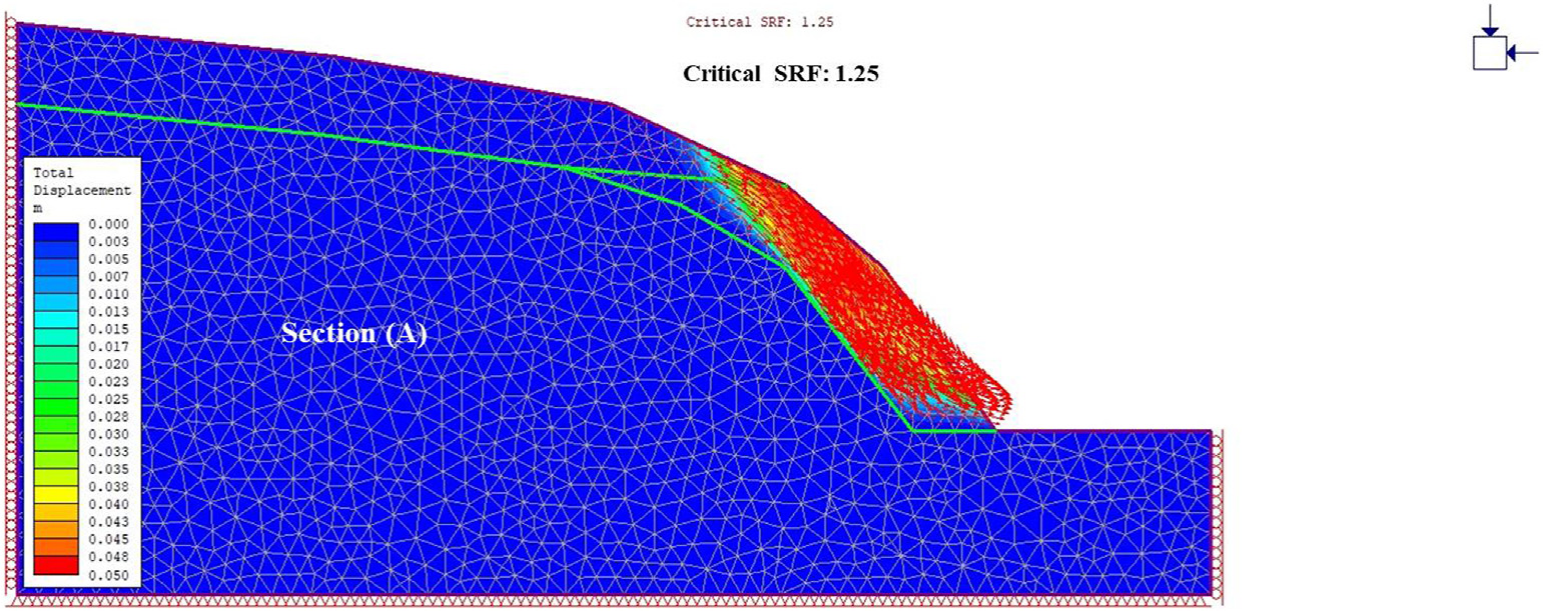
Contour of total displacement at section A.

**Figure 14:**
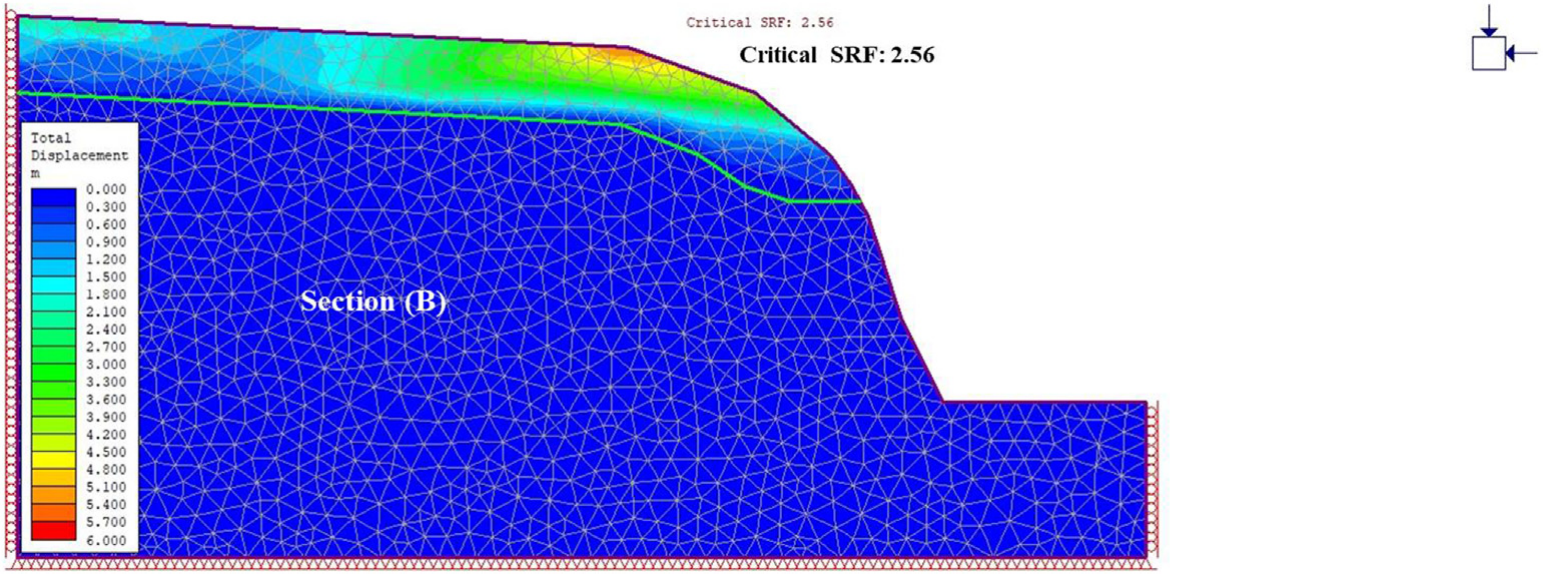
Contour of total displacement at section B.

A general overview of the studied slope is presented in Figure 6. The slope height was found to be 28 m with a slope angle ranging from 37° to 55° at section (A) (Figure 7) and 50°–75° at section (B) (Figure 8). The slope profile in section (A) is completely weathered (grade V) along with a cap of weathered materials (L1S1) overlying the slope surface (Figure 7) and the slope profile in section (B) belong to a moderately (grade III) to highly (grade IV) weathered rock mass characterised by the absence of debris on the slope surface (Figure 8). The numerical models of the slope at both sections A and B are presented in Figures 9 and 10 respectively. The presence of weathering materials overlying the slope at section (A) (Figure 7) is an indicator of instability problems. The shear strain dispersion within the simulated slope at section (A) and section (B) can be observed in Figures 11 and 12 respectively. The maximum shear strain contour in section (A) (Figure 11) clearly revealed the region of the slope where the sliding may occur. Such a slip surface observed at section (A), highlighted with a red dashed line, seems to be the consequence of the debris materials overlying the slope, as the shear strain is concentrated along with the interface between the rock mass and debris, representing the least resistant surface. It has been reported that weathering is one of the factors that lead to the reduction in the inherent shear strength of the rock mass, which represents the maximum shear stress that the geological formation can withstand under slope (Biondino et al., 2020; Conforti and Buttafuoco, 2017; Tran et al., 2019). Therefore, the presence of weathered materials above the slope profile section (A) indicates that there has been an alteration in the strength parameters of the slope material, such that failure is more likely in section (A). The critical SRFs of both sections (A) and (B) were found to be 1.25 and 2.53, respectively. As can be seen, the SRF was reduced by approximately 50%. Presumably, the higher SRF observed in section (B) is due to the fact that this section does not exhibit significant debris material on the slope surface (Figure 8). This result is in agreement with the case study conducted by Seyed-Kolbadi et al. (2019), which concluded that the FoS of a non-homogeneous slope covered by a weak layer is less than that of the homogenous one. The weak material on the slope surface at section (A) may be due to its geometry along with a lower overall slope angle unfavourable to surface drainage, which favours its degradation by mineral dissolution. Another factor may be the formation of secondary minerals that tend to alter the inherent shear strength of the slope-forming material (Che et al., 2012; Regmi et al., 2014; Tran et al., 2019), weakening its critical SRF. An SRF of 1.25 at the slope profile section (A) indicates that the slope is marginally stable and may fall under the influence of triggering events, such as heavy rainfall or long-lasting rainfall and seismic activity. For example, weathered products may reduce the down-water infiltration and retain the fluid, which increases the shear stress through the increase in the pore-water pressure, thereby resulting in slope instability (Nguyen et al., 2020). Migoń (2013) studied the weathering and hillslope development and highlighted that the steep slope surface that sheds water may be more resistant to weathering and failure, as the weathering processes are enhanced by the availability of water, which is detrimental to the shear strength reduction of the geomaterial (Pei et al., 2020). Furthermore, Ietto et al. (2018) reported a strong correlation between the degree of weathering and the slope gradient and indicated that gentle slopes are more vulnerable to weathering. This is in agreement with the slope profile section (B) (Figure 8), where the greater overall slope angle enhances the surface runoff (Pei et al., 2020) and may have contributed to the reduction in the alterability of the rock mass/strength factor, making it more stable than that in section (A) (Figure 7). The simulated total displacement obtained at section (A) (Figure 13) is 4.73 cm, which is approximatively a factor of 10 higher than that obtained in section (B) (Figure 14), equal to 0.46 cm, which shows the extent of the probable zone of failure in section (A) as compared with that in section (B).

## 5 Conclusions

Slope geometry plays a significant role in weathering processes. In this study, two different slope profile sections of the same slope following the bypass of the road were investigated. The rebound hardness value and other weathering indices associated with the field investigation were used to classify the weathering grade of each section. Section (A), with a relatively low overall slope angle, was completely weathered with a cap of debris, indicating a higher weathering rate at this section compared to the relatively steeper slope profile in the moderately to highly weathered section (B). The result of the numerical simulation at each section of the slope is based on the finite-element-method in the Phase2 software, generating an SRF of 1.25 in section (A) and 2.53 in section (B). The relatively low slope angle at section (A) enhances the weathering process, which is responsible for the alterability of the inherent shear strength of the slope-forming material, hence, promoting its instability.

## Declarations

### Author contribution statement

G. C. Komadja: Conceived and designed the experiments; Performed the experiments; Analyzed and interpreted the data; Contributed materials, analysis tools, or data; Wrote the paper.

S. P. Pradhan, A. Ratna Roul: Conceived and designed the experiments; Analyzed and interpreted the data; Contributed materials, analysis tools, or data; Wrote the paper.

B. Adebayo: Analysed and interpreted the data; Contributed materials, analysis tools, or data; Wrote the paper.

J. B. Habinshuti, L. Adissin Glodji: Contributed materials, analysis tools, or data; Wrote the paper.

L. Adissin Glodji: Contributed materials, analysis tools, or data; Wrote the paper.

A. P. Onwualu: Contributed materials, analysis tools, or data; Wrote the paper.

### Funding statement

This work was supported by the Pan African Materials Institute (PAMI) of the 10.13039/501100005749African University of Science and Technology (AUST) (AUST/PAMI/2015/5415-NG), and the World Bank in collaboration with the 10.13039/501100001409Department of Science and Technology (DST), Government of India (DST-1364-WRC a part of the DST-1264-AHC), which was coordinated by IIT Roorkee to strengthen African Centres of Excellence (ACEs) (https://www.iitr.ac.in/dstai/index.html) C.G. Komadja was awarded a scholarship by the African Development Bank (AfDB) to cover tuition fees and related study costs for the doctoral program at the African University of Science and Technology (AUST).

### Competing interest statement

The authors declare no conflict of interest.

### Additional information

No additional information is available for this paper.
